# A NLP-based semi-automatic identification system for delays in follow-up examinations: an Italian case study on clinical referrals

**DOI:** 10.1186/s12911-024-02506-2

**Published:** 2024-04-23

**Authors:** Vittorio Torri, Michele Ercolanoni, Francesco Bortolan, Olivia Leoni, Francesca Ieva

**Affiliations:** 1 MOX - Modelling and Scientific Computing Lab, Department of Mathematics, Politecnico di Milano, Piazza Leonardo da Vinci 32, Milan, 20133 Italy; 2 ARIA s.p.a - Azienda Regionale per l’Innovazione e gli Acquisti, Via Taramelli 26, Milan, 20124 Italy; 3 U.O. Osservatorio Epidemiologico, DG Welfare, Regione Lombardia, Piazza Città di Lombardia 1, Milan, 20124 Italy; 4 HDS - Health Data Science Centre, Human Technopole, Viale Rita Levi-Montalcini 1, Milan, 20157 Italy

**Keywords:** Natural language processing, Referrals, Follow-up examinations, Public healthcare system, Quality of healthcare

## Abstract

**Background:**

This study aims to propose a semi-automatic method for monitoring the waiting times of follow-up examinations within the National Health System (NHS) in Italy, which is currently not possible to due the absence of the necessary structured information in the official databases.

**Methods:**

A Natural Language Processing (NLP) based pipeline has been developed to extract the waiting time information from the text of referrals for follow-up examinations in the Lombardy Region. A manually annotated dataset of 10 000 referrals has been used to develop the pipeline and another manually annotated dataset of 10 000 referrals has been used to test its performance. Subsequently, the pipeline has been used to analyze all 12 million referrals prescribed in 2021 and performed by May 2022 in the Lombardy Region.

**Results:**

The NLP-based pipeline exhibited high precision (0.999) and recall (0.973) in identifying waiting time information from referrals’ texts, with high accuracy in normalization (0.948-0.998). The overall reporting of timing indications in referrals’ texts for follow-up examinations was low (2%), showing notable variations across medical disciplines and types of prescribing physicians. Among the referrals reporting waiting times, 16% experienced delays (average delay = 19 days, standard deviation = 34 days), with significant differences observed across medical disciplines and geographical areas.

**Conclusions:**

The use of NLP proved to be a valuable tool for assessing waiting times in follow-up examinations, which are particularly critical for the NHS due to the significant impact of chronic diseases, where follow-up exams are pivotal. Health authorities can exploit this tool to monitor the quality of NHS services and optimize resource allocation.

**Supplementary Information:**

The online version contains supplementary material available at 10.1186/s12911-024-02506-2.

## Background

In Italy, the National Health System (NHS) plays a crucial role in healthcare, covering a significant portion of healthcare expenditure (75.6% in 2021 [1]) and ensuring universal coverage for citizens. Given the profound impact of healthcare services on citizens’ quality of life and on the government budget, it is essential for public authorities to monitor the services provided by the NHS.

Chronic diseases exert a substantial burden on the health system, often requiring frequent follow-up examinations. The management of these examinations is often complex for both patients and General Practitioners (GPs) [2–4], but their inadequate provision can lead to increased rehospitalizations and worsened patient outcomes [5–14]. Moreover, several studies have examined the costs associated with chronic diseases, evidencing the cost-effectiveness of a timely provision of follow-up examinations [15–17].

The Italian NHS mandates a referral for follow-up examinations, that can be filled in by both GPs and specialized physicians. To guarantee appropriate waiting times, physicians are required to specify the priority of an examination in the referral. The standard referral form has four possible priority values (*U, B, D, P*), that correspond to 3, 10, 30-60 (depending on whether it is a specialist or instrumental examination) and 120 days of maximum waiting time, respectively [18]. While this priority mechanism has had an overall positive effect on the health care system [19], the standard priority classes inadequately capture disease-specific timing requirements for follow-up examinations. For this type of exams, physicians should select the default priority class (P) and manually specify the exact timing in the referral’s free-text field. Consequently, assessing whether a specific follow-up examination has been provided in a timely manner becomes challenging, resulting in the exclusion of follow-up examinations from waiting time monitoring as per the Italian National Plan for the Management of Waiting Lists [18].

To address this limitation and enable waiting time monitoring, it is necessary to analyze the unstructured textual field that contain temporal information. Automating this process requires the utilization of Natural Language Processing (NLP) techniques. NLP is a subfield of artificial intelligence at the intersection of computer science, statistics and linguistics [20, 21], dealing with the representation and analysis of textual data. In the healthcare domain, a vast amount of unstructured textual data exists, particularly in Electronic Health Records (EHRs) clinical notes. These texts contain valuable information that is only partially represented in the structured EHR fields [22]. In the last decade, NLP has begun to find applications in this domain, despite facing challenges such as the specific lexicon, high ambiguity, and numerous context-specific abbreviations [23]. However, a key limitation of NLP is that most tools and models are developed for English texts, even though recent years have seen an increase in NLP applications to other languages [24].

In this paper, we present a tailored NLP-based pipeline for extracting temporal information from the textual fields of Italian referrals. This tool aims to facilitate the monitoring of waiting times by healthcare authorities.

Our pipeline was applied to a dataset of referrals from the Lombardy Region, Italy’s most populous region, with 10 million inhabitants. Italy’s NHS administration is largely decentralized, with the Ministry of Health issuing laws and regulations while the 19 regions, plus the 2 autonomous provinces of Trento and Bolzano, bear responsibility for organizing, administering, and evaluating NHS services in their respective territories [25]. Therefore, our work is expected to pave the way for the definition of regulations regarding the indication and monitoring of waiting times for follow-up examinations in the Lombardy Region.

The data sources used in this work are sourced from administrative databases, which contain routinely collected data for operational purposes in the Lombardy Region. Although administrative databases offer comprehensive coverage of the population and do not require additional data collection costs [26], they do pose challenges related to data quality, source integration, and the lack of specific research design [27, 28]. Despite these challenges, administrative data have been extensively used for statistical analysis in the healthcare domain [29–32].

To the best of our knowledge, this is the first work applying NLP techniques on Italian referrals, using open-source software (Venturelli et al [33] applied NLP techniques to verify prescription appropriateness on similar data, but with proprietary software). Additionally, our work is the first to address the issue of waiting times for follow-up examinations in Italy, providing an essential tool for monitoring this critical indicator of healthcare service quality.

The rest of this paper is organized as follows: in Methods section we present the data and the methods adopted for our analysis, Results section shows the results, discussed, together with limitations and possible developments, in Discussion section, while Conclusions section contains conclusive remarks.

All analyses were conducted in Python [34] and the code is available by the authors upon request.

## Methods

In this section, we present the data utilized in our analysis (Data structure and contents section) and describe the pipeline we developed (Methods section).

### Data structure and contents

The dataset employed for this analysis consists of all referrals for specialists and instrumental examinations in Lombardy Region, prescribed in 2021 and performed up to May 2022.

The data have been obtained from two distinct sources:

1. *Anonymized Electronic Referrals*: this dataset includes information entered by the prescribing physician, such as the type of examination and the clinical question.
2. *Anonymyzed Performed Examinations*: this dataset contains data entered by healthcare facilities where the examinations are conducted. It includes information like the date of booking, the first date proposed to the patient, the actual examination date, and the healthcare facility details.

Both datasets contain records for individual prescribed examinations. However, since a referral can encompass multiple examinations (up to eight), some fields pertain to each individual examination (e.g., examination type, date performed), while others relate to the entire referral (e.g., referral date, physician’s ID), and these shared values apply to all records within the same referral.

Table 1 summarizes the variables used in our analysis.

**Table 1:** Description of the dataset

Variable	Source	Description
Anonymized Doctor ID	(1)	Unique ID of the prescribing physician
Anonymized Referral ID	(1)	Unique ID of the referral
Date of referral	(1)	Date when the physician filled out the referral
Date of booking	(2)	Date when the patient booked the examination
First proposed date	(2)	First date proposed to the patient by the booking system
Accepted date	(2)	Date accepted by the patient to perform the examination (may be later than the first proposed date)
Type of examination	(1)	Type of specialist or instrumental examination requested
O/Z Flag	(2)	Flag indicating if the examination is subject to waiting time monitoring (O) or not (Z)
Healthcare facility	(2)	Hospital or outpatient clinic where the examination is performed
ATS (LHA - Local Health Authority)	(1)	LHA of the prescribing physician
Clinical question	(1)	Free text containing the reason for the referral and possibly its timing

List of variables included in the analyzed dataset, with source and description. Sources: (1) = Anonymized Electronic Referrals, (2) = Anonymized Performed Examinations

Since both physician and referral IDs have been replaced with anonymous identifiers, no sensitive information is present in the structured data. The free text field does not include sensitive information, since it is used only to report information about the reasons behind the referral and the time for follow-up examinations. In any case, to make sure that we do not fall under the scope of the GDPR (General Data Protection Regulation), a preliminary check of the presence of sensitive information (e.g., names, phone numbers, SSN identifiers) has been performed by Lombardy Region, removing them from texts, where present. The procedure is detailed in Additional file 1, Section D.

Exclusion criteria were applied to remove referrals not relevant for waiting time monitoring: screenings, emergency room (ER) visits, urgent referrals (labelled with priority class *U*, typically necessitating ER visits), and lab tests (which do not require booking in Lombardy).

Of the variables shown in Table 1, the most delicate is the O/Z flag. It has the value Z for all the types of referrals that are not subject to the monitoring of waiting times, which currently include follow-up examinations, but also a few other types of referrals, in particular psychiatry, sports medicine and dialysis, for which all the referrals, independently from being follow-up or first exams, should have the Z flag. While for specialist examinations there are two codes for each type of specialist examination, one corresponding to first access and one to follow-up, instrumental examinations have a unique code for each type of exam, so this flag is the best proxy to assess if an instrumental examination is a follow-up or not. Considering that our analysis of instrumental examinations will be focused on radiological exams, we do not expect to have a relevant number of them with the Z flag without being a follow-up examination. Both in our analysis of the full dataset and in selecting the data for the “training” and test set for the pipeline, we use follow-up codes for specialist examinations and the Z flag for radiology examinations.

Regarding the type of examination, the dataset encompasses 1304 different types of examinations. Among them, the 59 specialist examination types and the 130 radiology exam types (MRIs, CTs, X-rays, ultrasounds, and mammography scans) are the most relevant ones due to their inclusion in the priorities for waiting times set by the Lombardy Region [35].

The focus of our analysis is on the free text field (*Clinical Question*), which has a value in 99.9% of referrals. Table 2 provides examples of its values along with their English translations.

**Table 2:** Examples of clinical questions

Clinical question (ITA)	Clinical question (ENG)
- follow up in Ca endometroide	- follow-up in endometroid carcinoma
- CONTROLLO IN PREGRESSA POLIPECTOMIA	- MONITORING IN PREVIOUS POLYPECTOMY
- regolarizzazione 4 dito mano dx	- regularisation 4th finger right hand
- ca mammario in follow up; scan osseo 07 20 neg; dolore dorso-lombare dndd che si irradia a sinistra	- breast carcinoma in follow up; bone scan 07 20 neg; dorso-lumbar pain of nature to be determined radiating to the left
- Dermatite	- Dermatitis
- diabete mellito ottobre 2021	- diabetes mellitus October 2021
- sarcoma di ewing emibacino sx	- ewing’s sarcoma left haemibacinus
- F.A. CRONICA	- CHRONIC ATRIAL FIBRILLATION
- 01282 - Altre forme di tubercolosi respiratoria, risultato dell’esame batteriologico o istologico non conosciuto (allo stato attuale	- 01282 - Other specified respiratory tuberculosis, bacteriological or histological examination unknown (at present
- per ter apia valutazione qt	- for ter hapy qt evaluation

Examples of clinical questions from the dataset. Italian abbreviations are expanded in the English translation

### Methods

The extraction of temporal information from text has been extensively studied in the literature [36], with rule-based and hybrid approaches being commonly used.

These approaches make extensive use of regular expressions, a powerful NLP technique that allows the definition of a formal language using specific symbols and patterns [37]. This language defines the set of strings that conform to the specified expressions. The main advantages of using regular expressions for information extraction is that they do not require a large annotated training set and they are a white-box system, overcoming the explainability challenges associated with many machine learning and deep learning models [38]. However, existing tools for extracting temporal information have limitations in terms of language and domain coverage. Most tools are initially developed for English and subsequently extended to other languages. Among these tools, HeidelTime [39] is the only one that currently covers Italian [40]. Another limitation is the domain-specific nature of temporal information, with expressions varying depending on the specific domain. In the medical domain, there are often abbreviations and particular temporal expressions that may not be adequately covered by general-purpose tools. Notably, the i2b2 challenge in 2012 was centered around identifying temporal relations in English clinical texts, with the winning team utilizing regular expressions [41]. While some tools exist for extracting temporal information from clinical texts [42], none of them currently cover the Italian language.

To address these limitations we developed a pipeline specifically tailored for extracting temporal information from Italian referral texts.

**Fig. 1:**
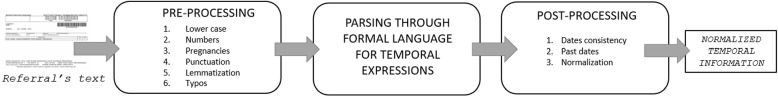
Schema of the pipeline for the extraction of temporal information from textual fields of referrals

The pipeline proposed in this paper is depicted in Fig. 1. It encompasses three key steps:

1. Pre-processing: the referral text undergoes a pre-processing step aimed at simplifying the text, enabling subsequent steps to be more effective.
2. Parsing: the pre-processed text is parsed using a formal language to extract temporal indications related to waiting times.
3. Post-processing: the extracted temporal indications are normalized, resulting in the production of Normalized Temporal Information (NTI). This allows for the computation of delays and facilitates further analysis.

In our pipeline, the core component is the parsing step, which employs a formal language defined through nested regular expressions to capture various types of temporal indications. We implement this step using the *reparse* Python library [43], facilitating the definition of a formal language using regular expressions.

This formal language for temporal expressions is developed based on a manually annotated dataset of 10 000 clinical questions randomly selected from follow-up examinations. The relatively large size of the annotated dataset is due to the low frequency of temporal indications observed during the annotation process.

Using these annotations, the formal language is developed and validated on another dataset of 10 000 manually annotated texts. The language incorporates rules for different types of temporal indications identified during the annotation process, as well as common elements shared among multiple types for temporal indications (e.g.: a *time unit* can be used in both an *interval of time* and a *precise time*). More details on the annotation procedure, the test set and the formal language can be found in Additional file 1, Sections A, B and C, respectively.

In addition to parsing, our pipeline includes pre and post-processing steps to address specific cases and mitigate challenges such as misspelt words. Pre-processing includes lower-casing, transforming literal numbers into digits, removing confounding elements related to pregnancies, partial deletion of punctuation (excluding punctuation symbols used in dates), lemmatization using the Spacy Python library, and partial correction of typos. Stop words, often removed in NLP pre-processing [44], are retained in our pipeline as they provide essential context for understanding temporal relations. Lemmatization [45] helps normalize temporal-related terms, reducing the need for explicit handling of these cases in the formal language. More details on pre-processing can be found in Additional file 1, Section D.

Post-processing steps allow the completion of some missing information, the deletion of inconsistent values and the normalization of the extracted information. They are detailed in Additional file 1, Section E.

Once these steps are performed, we are able to quantify the delay, since it is possible to compare the extracted *NTI* with the first date proposed to the patient by the booking system.

**Fig. 2:**
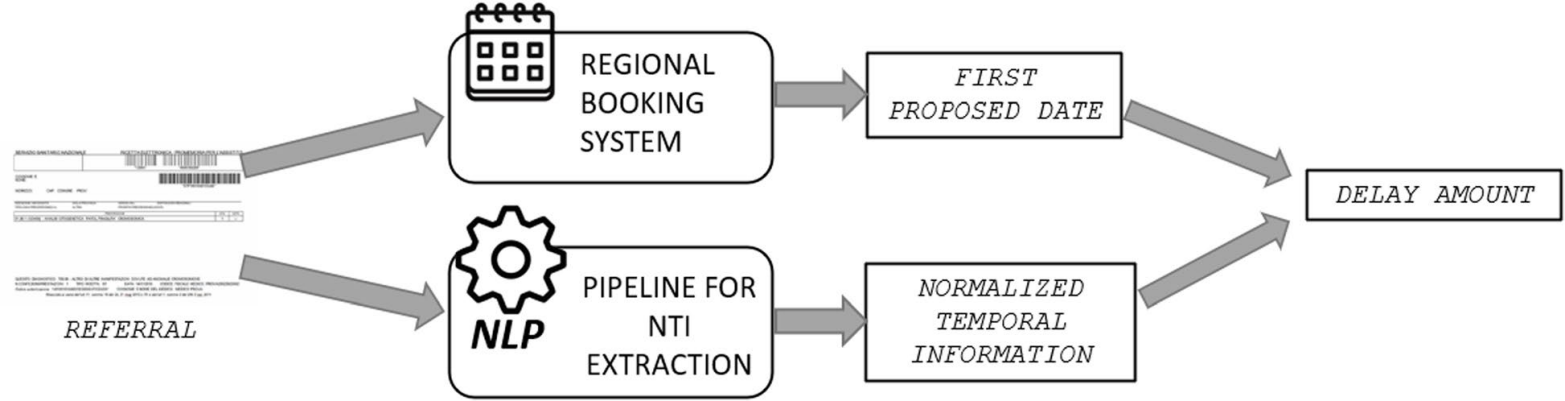
Schema of the procedure for the computation of delays for follow-up examinations

When a patient needs to book an examination, the Lombardy Regional Booking System proposes the available slots in a selected province and he/she can select among them. For the computation of delays, we consider as starting time the time of booking, that is when the patient books the exam As the date on which the exam has been performed, we consider the first proposed date, i.e., the date of the first slot that was proposed to the patient when he/she booked the exam. The NHS cannot be held responsible for delays resulting from late booking or choice of a later date by the citizen. Figure 2 summarizes the procedure for the computation of delays.

## Results

In this section, we present the results of our analysis. In particular, in Pipeline for the NTI extraction section we present the results for the pipeline on the manually annotated test set and in Temporal information and delays on the entire dataset section we present the outcome of the analysis on the full 2021 dataset.

### Pipeline for the NTI extraction

The pipeline’s evaluation is conducted on two levels: the identification of temporal information presence/absence (a binary classification problem) and the correctness of the extracted Normalized Temporal Information (NTI). The accuracy of the NTI extraction is assessed on both the entire test set and the subset containing temporal information to avoid bias caused by the low frequency of temporal information. The extraction results are presented in Table 3.

**Table 3:** Results of the pipeline on the test set for the temporal information extraction

Metric	Value
**Presence/Absence of the temporal indication**	
Accuracy	0.999
Precision	0.990
Recall	0.973
**Correctness of the** ***NTI***	
Accuracy on all referrals	0.998
Accuracy on referrals with any temporal indication	0.953
Accuracy on referrals with a follow-up temporal indication	0.948

Results of the pipeline on the test set, measuring different metrics with respect to both the binary classification problem of presence/absence of the temporal indication and to the accuracy of the extracted NTI

Analyzing the errors in the test set of 10 000 examinations, we observe 8 (0.08%) false negatives (FN), 3 (0.03%) false positives (FP), and 7 (0.07%) indications with incorrect values. False positives are mainly caused by expression ambiguities and abbreviations, while false negatives result from uncorrected misspellings and unaccounted expressions such as “today”. The extraction of incorrect values is primarily due to misspellings and multiple temporal indications within a single clinical question. Additional file 1, Section F, provides a detailed analysis of false positives, false negatives, and wrong values.

### Temporal information and delays on the entire dataset

At the end of this procedure, the dataset consists, for the year 2021, of 14 502 861 records, corresponding to 12 035 177 unique referrals. Within this dataset, there are 24 736 physicians, with 71% being specialized physicians and the remaining being GPs.

Applying the pipeline to the entire dataset enables the insertion of new variables related to follow-up examinations: the value and unit of measure of the extracted NTI. Among all Z referrals, 1.18% contain temporal indications. This percentage increases to 2.02% for follow-up specialist examinations and 2.16% for radiological exams with the Z flag.

Analyzing the presence of temporal information based on the type of prescribing physician reveals that 0.43% of Z referrals from GPs have a temporal indication, while this percentage increases to 2.38% for referrals from specialized physicians.

**Fig. 3:**
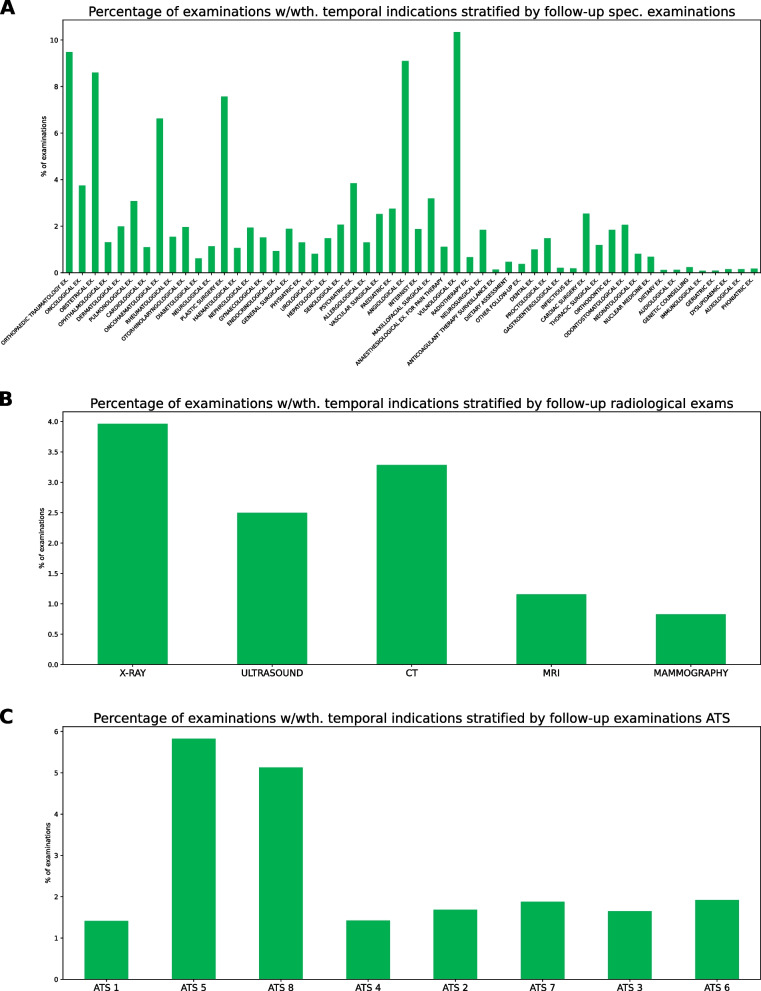
Presence of the temporal indication for follow-up examinations by type of specialist examination, type of radiological exam and ATS (LHA). Only examinations with $> 1000$ records are included

Figure 3 shows the presence of temporal information, stratified by different types of specialist examinations, radiological exams, and the different ATS (LHA) of prescribing physicians. Notable findings include a high percentage number (9.5%) of orthopaedic examinations reporting temporal indications. Being orthopaedic examinations very common, these imply they have by large the highest absolute number of temporal indications (21 298). Other exams with high percentage numbers correspond to lower absolute numbers: vulnological (10.5% - 262), angiological (9.1% - 376), obstretical (8.6% - 6 196), plastic surgery (7.6% - 1 325) and oncohaematological (6.6% - 1 671).

Among the 102 000 temporal indications that are extracted, the average delay is $-39$ days, indicating that, on average, the examinations are performed in a timely manner. The median delay is $-5$ days, with an interquartile range of 43 days, suggesting the presence of outliers. Among the subset of examinations with identified temporal indications, 16% exhibit delays, with an average delay of 19 days (SD=34). The median delay is 6 days, with an interquartile range of 17 days.

**Fig. 4:**
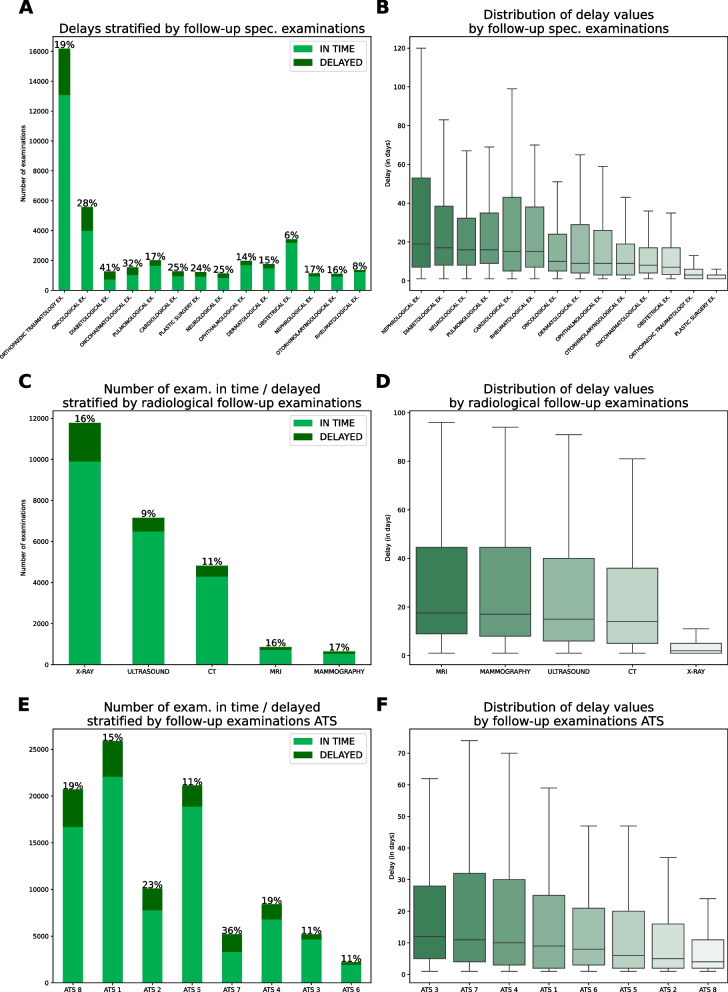
Delays (Delays are calculated as [*NTI*] – [time from date of booking to date proposed by the booking system to patient]) for delayed exams and amount of delayed exams by type of examination, type of radiological exam and ATS (LHA). Boxplots are ordered by median, while barplots are ordered by the absolute number of delayed exams. Only examinations with $> 1000$ records are included

Figure 4 provides a visual representation of the delayed follow-up referrals, stratified by type of specialist examination, type of radiological exam, and ATS (LHA). Additionally, boxplots illustrate the distribution of delays for the delayed exams.

In medical examinations, there are evident differences between various types of specialist examinations in terms of the number of delayed examinations and the extent of delays. The highest percentages of delayed examinations can be observed in diabetology (41%), oncohaemaotology (32%) and oncology (28%). Considering the entity of the delays, the highest delays are observed in nephrology (median 19 days), followed by diabetology (median 17 days) and pulmonology (median 16 days).

Regarding radiological follow-up exams, higher percentages of delays are observed in mammographies (17%), MRIs (16%), and X-rays (16%), with lower delays in MRIs compared to other radiological exams (median 2 vs 14-17 days).

Variations in delays are evident across different ATS (LHA). ATS 7 has the highest percentage of delayed follow-up examinations (36%), while ATS 11 exhibits the best performance.

## Discussion

This paper aims to analyze texts of Italian referrals to investigate waiting times for follow-up examinations. To the best of our knowledge, this is the first attempt in this direction.

The proposed approach has evident strengths and potential. The developed pipeline has proved to be able to extract and normalize temporal information from these texts with high accuracy, on both the recognition of the presence of temporal information and the correctness of the extracted normalized temporal information. One of the significant advantages of our pipeline is its inherent explainability. Unlike many machine-learning and deep-learning-based models, the rationale behind the system’s output is transparent and understandable. This attribute is crucial when a model is intended for use by health authorities. It is worth noting that this achievement was accomplished despite having used as a “training set” only a small percentage of the total available records. As we look forward, the availability of a larger annotated dataset will enable further fine-tuning of the system. While some extensions may be required to cover previously unseen types of temporal expressions, the pipeline’s architecture, based on a formal language, facilitates the introduction of new rules or extensions to existing ones.

Executing the pipeline when the majority of referrals for follow-up examinations contain a temporal indication will allow precise monitoring of the quality of follow-up examinations, in terms of waiting times. Manual analysis of this magnitude would entail an unsustainable cost for the NHS, estimated at over 30 000 hours of work, assuming 10 seconds per referral.

Limitations should also be acknowledged. In particular, it is essential to remark that the results regarding delays, including variations among geographical areas and medical disciplines, presented here serve as examples of potentially valuable insights for decision-makers. However, these findings should be validated in subsequent analyses when a more significant proportion of referrals will include timing indications.

Another limitation is related to the fact that we considered as follow-up instrumental examinations all instrumental examinations with the Z flag since it is the best proxy to identify follow-up instrumental exams, but Z referrals may also include other types of referrals, and follow-up referrals might not always be marked as Z. This should not particularly affect the radiological exams we analyzed, but it might affect other types of instrumental examinations.

Moreover, in the computation of delays in the provision of follow-up examinations, we considered the time between the first date proposed to the patient by the booking system and the date of booking. This implies excluding the impact of the following elements:

1. the time that might elapse between the request of a follow-up examination communicated by the specialist physician to the patient and the filled-out of the corresponding referral by the patient’s GP, when the specialist physician does not directly fill it out
2. the time between the date of the referral and the date of booking
3. the geographical position of the healthcare facility where the first slot proposed to the patient is located

We do not have access to data which allow quantifying the impact of the first and the third point, and we did not consider the second point since the focus of this analysis is the monitoring of the delays for which the NHS can be considered responsible. We report in Additional file 1, Section G, plots describing the number and the amount of postponed follow-up examinations. Different types of examinations show differences in both the number of postponed exams (from 29% of diabetological exams to 4% of oncoematological exams) and the amount of postponement. Some types of exams, such as orthopaedics, have shorter postponements. This can be expected due to the higher urgency of certain diseases, which makes patients less likely to postpone their follow-up exams with respect to other types of diseases, such as diabetes and cardiological ones, that might remain more stable and less urgent for long periods of time. Relevant differences can also be observed among different ATSs: ATS 1 has 29% of postponed examinations, while they are $< 1\%$ in ATS 4. This is probably due to the fact that certain ATSs cover more extensive territories, with more hospitals where the first proposed date could be offered, potentially far from patients’ residency. The numbers reported for the postponements increase the effective delay experienced by citizens, despite this is not taken into account by the quality metrics currently in use in the Italian NHS. Future analyses could better assess the reasons behind these postponements, which might lead health authorities to intervene both on the geographical factor and on patient awareness of the importance of in-time follow-up examinations for chronic diseases.

Among the possible extensions of our pipeline, we could enhance the recognition of typing mistakes, as they are prevalent in these texts. Additionally, we could consider incorporating the capability to associate temporal indications with specific examinations in cases where referrals contain multiple examinations with multiple temporal indications, all reported within the clinical question of the referral.

## Conclusions

NLP and textual analysis represent promising tools for enabling semi-automatic performance monitoring in public administration. In particular, in this paper, we have placed our focus on the analysis of health referrals, in order to monitor the waiting times for follow-up examinations.

Follow-up examinations constitute a substantial portion of the services provided by the NHS, and their impact is particularly significant, especially when considering chronic diseases. Consequently, they can no longer be disregarded in the assessment of the quality of NHS services. Mandating physicians to include time indications in referrals for follow-up examinations would empower healthcare authorities to proactively allocate resources and intervene when necessary. This proactive approach would ensure the timely and effective delivery of care, supported by data that would be impractical to produce through manual analysis due to the extensive time it would require.

In summary, the application of NLP and textual analysis in monitoring follow-up examinations can contribute to reducing healthcare burdens, improving patient well-being, and optimizing the allocation of resources within the NHS.

### Supplementary Information

**Additional file 1: Supplementary Material.** Contains more details about the pipeline, the dataset and the error analysis.

## Data Availability

The data that support the findings of this study are available from Regione Lombardia but restrictions apply to the availability of these data, which were used under license for the current study, and so are not publicly available. Data are however available from the authors upon reasonable request and with permission of Regione Lombardia.

## Abbreviations

NHS: National Health System
NLP: Natural Language Processing
GP: General Practitioner
EHR: Electronic Health Record
ER: Emergency Room
ATS/LHA: Agenzia di Tutela della Salute / Local Health Authority
GDPR: General Data Protection Regulation
SSN: Social Security Number
SD: Standard Deviation
IQR: Interquartile Range
MRI: Magnetic Risonance Imaging
CT: Computerized Tomography
NTI: Normalized Temporal Information
FP: False Positive
FN: False Negative

## Availability of data and materials

The code is available by the authors upon request.
