# Supplementary material for: A NLP-based semi-automatic identification system for delays in follow-up examinations: an Italian case study on clinical referrals
Source: BMC Med Inform Decis Mak. 2024 Apr 23;24:107. doi: 10.1186/s12911-024-02506-2 (PMC11040851; doi:10.1186/s12911-024-02506-2)
Supplement: Supplementary file 1 — Additional file 1: Supplementary Material. Contains more details about the pipeline, the dataset and the error analysis. [file 12911_2024_2506_MOESM1_ESM.pdf]

Appendix A Annotation procedure

The manual annotation process for both the "training" and the test set has been performed by initially assigning to each text a binary indication of the presence/absence of the temporal indication. Subsequently, all the texts in which a temporal indication has been recognized have been labelled with the following elements:

1. a binary indication for the temporal information to be referred to the date of the next follow-up examination;
2. the portion of the string containing the temporal indication;
3. a normalized value of the temporal indication (dates in the format *dd-mm-yyyy*, other indications as numeric values);
4. the unit of measure of the normalized value (e.g., date, month, week);
5. the type of temporal indication (e.g., *Da* (Since), *Tra* (In), *Data completa* (Full date));

This process has been repeated three times and discrepancies have been manually analyzed to reduce the probability of errors in the annotations.

Appendix B Test dataset

The manually annotated test set consists of 10 000 clinical questions selected randomly among follow-up examinations. Details on the annotations are reported in Table B1.

Table B1 Types of temporal information in the manually annotated test set

Type of indication	N. of records
**Follow-up timings**	**291**
Tra (In)	134
A (in) + amount of time or month	32
Per (for) + month	5
Alla x settimana (at the x-th week)	2
Dopo (After) + amount of time	1
Entro (within) + amount of time	6
Time indication (e.g., *2 months*, *October*)	30
Precise date	81
**Other timings**	**27**
Past exams	16
Week of pregnancy	5
Pregnancy termination date	1
Date of last period (for pregnancy)	1
Date of origin of symptoms/disease	4

Analysis of the different types of temporal indications manually annotated in the test set, including temporal indications not referred to waiting times

Appendix C Formal language for temporal information

The formal language we developed to extract temporal information is reported here, using the notation of formal grammars.

```
TEMPORAL_INDICATION →CONTROL_INDICATION | DA | FA

CONTROL_INDICATION →FUTURE_DISTANCE | IL | FULL_DATE |
    ↪ IMPRECISE_FUTURE

SPACES →\s SPACES | ϵ

FUTURE_DISTANCE →INTERVAL_FUTURE_DISTANCE |
    ↪ PRECISE_FUTURE_DISTANCE

INTERVAL_FUTURE_DISTANCE →NUMBER SPACES SEPARATOR SPACES NUMBER
    ↪ SPACES TIME_UNIT

NUMBER →[0-9]+

SEPARATOR →- | /

TIME_UNIT →DAY_UNIT | WEEK_UNIT | MONTH_UNIT | YEAR_UNIT

DAY_UNIT →gg | giorno

WEEK_UNIT →sett | settimana

MONTH_UNIT →mese

YEAR_UNIT →anno

PRECISE_FUTURE_DISTANCE →NUMBER SPACES TIME_UNIT

IL →il SPACES (FULL_DATE | DAY_MONTH_DATE | MONTH_YEAR_DATE | DAY)

FULL_DATE →DAY DATE_SEPARATOR MONTH DATE_SEPARATOR YEAR

DAY →([0-3][0-9]) | [0-9]

DATE_SEPARATOR →- | / | . | +

MONTH →NUMERICAL_MONTH | LITERAL_MONTH

NUMERICAL_MONTH →(1[0-2]|0[1-9]|[1-9])
```

```
LITERAL_MONTH →SHORT_LITERAL_MONTH | FULL_LITERAL_MONTH

SHORT_LITERAL_MONTH →gen|feb|mar|apr|mag|giu|lug|ago|set(?!timana)
    ↪ |sett(?!imana)|ott|nov|dic

FULL_LITERAL_MONTH →gennaio|febbraio|marzo|aprile|maggio|giugno|
    ↪ luglio|agosto|settembre|ottobre|novembre|dicembre

YEAR →FULL_YEAR | SHORT_YEAR

FULL_YEAR →20[0-9][0-9]

SHORT_YEAR →[0-9][0-9]

DAY_MONTH_DATE →DAY DATE_SEPARATOR MONTH

MONTH_YEAR_DATE →MONTH DATE_SEPARATOR YEAR

IMPRECISE_FUTURE →DAY_MONTH_DATE | MONTH_YEAR_DATE | LITERAL_MONTH

DA →(da|dal) SPACES FUTURE_DISTANCE

FA →FUTURE_DISTANCE SPACES fa
```

Appendix D Pre-processing steps

To ensure the absence of sensitive information of both doctor and patients in the free text fields, texts have been pre-filtered with the following regular expressions:

```
# A name (from predefined list of names) and surname (assuming the
    ↪ word after the name is a surname)
'\b(' + r'|'.join(names) + r')\b\s*[a-z]+\b\s*'

# A title like doctor, professor, followed by a surname (assuming the
    ↪ word after the title is a surname)
'\b(dott|dottor|dr|dot|dottoressa|dottssa|dssa|dotssa|drssa|sig|signor
    ↪ |signora|sig\.?ra|sig\.?na|signorina|prof|prof\.?ssa|professor|
    ↪ professoressa)\.?\b\s*[a-z]+\b\s*'

# An Italian SSN (Codice Fiscale)
'\b[a-z]{6}\d{2}[a-z]\d{2}[a-z]\d{3}[a-z]\b'

#Italian phone numbers
```

```
'\b((3\d{2}[\s|\-|\.|\/]?\d{7})|(0\d[\s|\-|\.|\/]?\d{8}) | (0\d{2}[\s
    ↪ |\-|\.|\/]?\d{7})|(0\d{3}[\s|\-|\.|\/]?\d{6}))\b'
```

Before running our parser we then performed the following operation:

- replace all numbers expressed in words with their corresponding digits
- remove all expressions of the type `\d+ \s* settimana` (*n-th week*) in sentences where a pregnancy is mentioned
- remove all dates that immediately follow the word `gravidanza` (*pregnancy*) or the expression `termine il` (*termination on*)
- for punctuation removal, remove all punctuation symbols present in `string.punctuation` from the Python library `string`, except for the following characters: `./()-`, since they are commonly used in dates.
- to correct some typos, replace all the words with less than 5 occurrences in the dataset that have a similar word with more than 5 occurrences. The similarity is computed with the `SequenceMatcher.ratio(s1,s2)` function, which corresponds to $2M/T$, where M is the number of matching characters between $s1$ and $s2$ and T is the total number of characters in $s1$ and $s2$. The threshold is set to 0.8.
- to simplify the grammar, lemmatize words using the *Spacy* Python library, with the model *it_core_news_sm*

Appendix E Post-processing steps

The *reparse* library allows defining functions to be executed during the grammar parsing, i.e. during the process in which a string is analyzed to understand if it belongs or not to the language defined by the grammar. These functions act as post-processing functions (corresponding to the third block of Figure 1 of the main manuscript), and we defined them so as to normalize the representation of the extracted temporal information, making it possible to use this information to compute delays. We consider two possible types of normalized formats: dates and amounts of time. Dates are all transformed in the standard format *dd-mm-yyy*, while amounts of time have a numeric value and a unit of measure (*days, weeks, months* or *years*).

The post-processing also completes missing information, considering the referral date. The rules used to complete missing information are reported in Algorithm 1. It should be noted that when a type of temporal indication is part of another (e.g., `LITERAL_MONTH` in `FULL_DATE`) the completed information can be overwritten by the container information (e.g., the year will be the one reported in the `FULL_DATE`, not the one completed in the `LITERAL_MONTH`). At this point, we also discard all the dates that are not consistent (e.g., *31.06*, since the day *31* is not present in the month *06*). Doing this only at post-processing allows to avoid an explicit specification of all the particular date cases in the grammar.

Eventually, all dates that are prior to the referral date are discarded.

Algorithm 1 Rules to complete missing information in extracted time indication

$ref\_month \leftarrow referral.referral\_date.month$
$ref\_day \leftarrow referral.referral\_date.day$
$ref\_year \leftarrow referral.referral\_date.year$
$time\_ind \leftarrow extract\_time\_indication(referral.clinical\_question)$

if $type(time\_ind)$==`MONTH_YEAR_DATE` or $type(time\_ind)$==`LITERAL_MONTH` **then**
    $time\_ind.day \leftarrow get\_last\_day\_of\_month(time\_ind.month)$
end if

if $type(time\_ind)$==`IL` and $isna(time\_ind.month)$ **then**
    if $time\_ind.day \leq ref\_day$ **then**
        $time\_ind.month \leftarrow (ref\_month + 1)\%12$
    else
        $time\_ind.month \leftarrow ref\_month$
    end if
end if

if $type(time\_ind)$==`DAY_MONTH_DATE` or $type(time\_ind)$==`LITERAL_MONTH` or ($type(time\_ind)$ == `IL` and $isna(time\_ind.year)$) **then**
    if $time\_ind.month \leq ref\_month$ or ($time\_ind.month == ref\_month$ and $time.day \leq ref\_day$) **then**
        $time\_ind.year \leftarrow ref\_year + 1$
    else
        $time\_ind.year \leftarrow ref\_year$
    end if
end if

Appendix F Errors analysis

In this section we perform a detail reporting of the errors registered on the test set by the NTI extraction pipeline.

The false positives are the following values:

1. *recidiva di panniculite (dopo un mese di augmentin) - recurrence of panniculitis (after one month of augmentin)*: probably referred to a previously prescribed medication and not to the fact the next exam should be done after one month of augmentin
2. *frattura biossea 1/4 distale avambraccio destro - right forearm distal 1/4 bony fracture*: *1/4* is not a date here
3. *allergia a sedano, dic in rivalutazione - allergy to celery, irritative contact dermatitis under reevaluation*: here the problem is in the acronym *dic*, which is often used as an abbreviation of the month December (*Dicembre*) in Italian, but in this case, it stands for *dermatite irritativa da contatto (irritative contact dermatitis)*

The first one is the most difficult to correct since the sentence has a degree of ambiguity and could be even interpreted in the sense of waiting one month before doing the next examination. The second one is referred to orthopaedics and may suggest inserting specific rules for these cases. The last one is determined by the usual problem of ambiguity in abbreviations. It is difficult to avoid since even in the field of allergology *dic* could be used to indicate *december*.

The false negatives are the following ones:

1. *controllo in tx fegato < 1 annoMGEINF-N6prelievo in ambulatorio infermieristico - control in liver tx < 1 yearMGEINF-N6drawal in nursing clinic*: the problem is that the word *anno (year)* is not separated from the following one and consequently it is not recognized
2. *fr 3 mtc sx tr auna settimana ca - fr 3 mtc sx i none week ca*: the problem is that the word *tra (in)* has one letter attached to the next word *una (one)*, so the latter is not recognized
3. *ipotiroidismo cod 191 - tra 2 mesi- - hypothyroidism cod 191 - in 2 months-*: the problem is related to the dash attached to *mesi (months)*, which causes an incorrect lemmatization
4. *retinopatia diabetica, AMB.CHIRURGIA COMPLESSA PER OGGI - diabetic retinopathy, COMPLETE SURGERY AMB.SURGERY FOR TODAY*: we did not consider *today* among the time indications, since it is anomalous that a follow-up examination is requested for today
5. *k mammella operato, pregresso LH, controllo tra circa 6-7mesi - k breast operated, previous LH, check in about 6-7months*: the absence of a space in *6-7mesi (6-7months)* causes a wrong lemmatization
6. *sospetta DAC - controllo per lettura patch dopo circa 48 ore - suspected DAC - check for patch reading after about 48 hours*: we did not include temporal indication expressed as hours, since it seemed to be anomalous for follow-up examinations, but this suggests its inclusion
7. *controllo il gg11 Lombalgia - check on day11 Lumbago*: the problem is due to the absence of a space between gg and 11
8. *ciclo di 8 ctr in D Umore c/o CTDD (95B002) dal 2022 - cycle of 8 ctr in D Mood c/o CTDD (95B002) from 2022*: here the indication is related to the starting time of the follow-up examinations, and not to the time within which they should be performed, so it is not really a false negative

While it is possible to fix the problems related to dashes attached to words, which cause incorrect lemmatization, it is harder to detect missing spaces.

The texts from which the pipeline extracted a wrong values are the following ones:

1. *reflusso acido - cerume periodico / per Luglio-Agost - acid reflux - periodic cerumen / for July-August*: the pipeline returns 31/07/2021 instead of 31/08/2021, due to a missing letter in *Agosto (August)*
2. *Controllo ad 1 mese e gispotomia in frattura biossea avambraccio dx (chirurgia il 15/3/21) - 1-month check-up and gispotomy in right forearm bony fracture (surgery on 15/3/21)*: the pipeline returns 1 month, which is the most correct value. The labelling included also 15/3/21, since it is still a date of a future examination

3. *ulcera gamba dx-visita fine agosto e fine settembre 2020 - ulcer right leg-visit end of August and end of September 2020*: the pipelines returns 31/08/2021, which is wrong, since both September and August probably refers to 2020, even if it is not completely unambiguous
4. *frattutra base V MTT piede sinistro 7 giorni fa. In sala gessi 3 set - fracture base V MTT left foot 7 days ago. In plaster room 3 set*: the pipeline returns 3/09/2021, but the label is 3 weeks. The text is in effect ambiguos, since *set* is an abbreviation for both September and week, in Italian.
5. *controllo dopo rimozione di collare ecrvicale in trauma distorsivo rachide cervicale: visita da programmare per il 24-25 di giugno - check after removal of ecrvical collar in cervical spine distortion injury: visit to be scheduled for 24-25 June*: the pipeline returns 30/06/2021 instead 26/06/2021, due to the double number 24-25 which is not recognized
6. *k sene V8901 per dicembre 21- genaioo 2022 - k breast V8901 for December 21-January 2022*: the pipeline returns 31/12/2021 instead of 31/01/2022, due to the mispelling of *gennaio (January)*
7. *fratt tibia distale destra - medicazione giovedi 25/11 - RX e visita a 30 gg - right distal tibia fracture - dressing Thursday 25/11 - X-ray and examination at 30 days*: the pipeline returns 25/11/2021, which is correct, but there is also the examination at 30 days

Some of these wrong values can be fixed, like the errors due to multiple days separated by dashes. The most complex situation is when there is more than one indication since it is not enough to recognize them all, but it should be understood which is the correct one for the examination that is being considered.

Appendix G Postponments

Figure G1 described the number of postponed examinations and the amount of postponments by type of examination and ATS.

Appendix H Number of examinations

Figure H2 described the number of examinations by type of specialist examinations, radiological examinations and ATS.

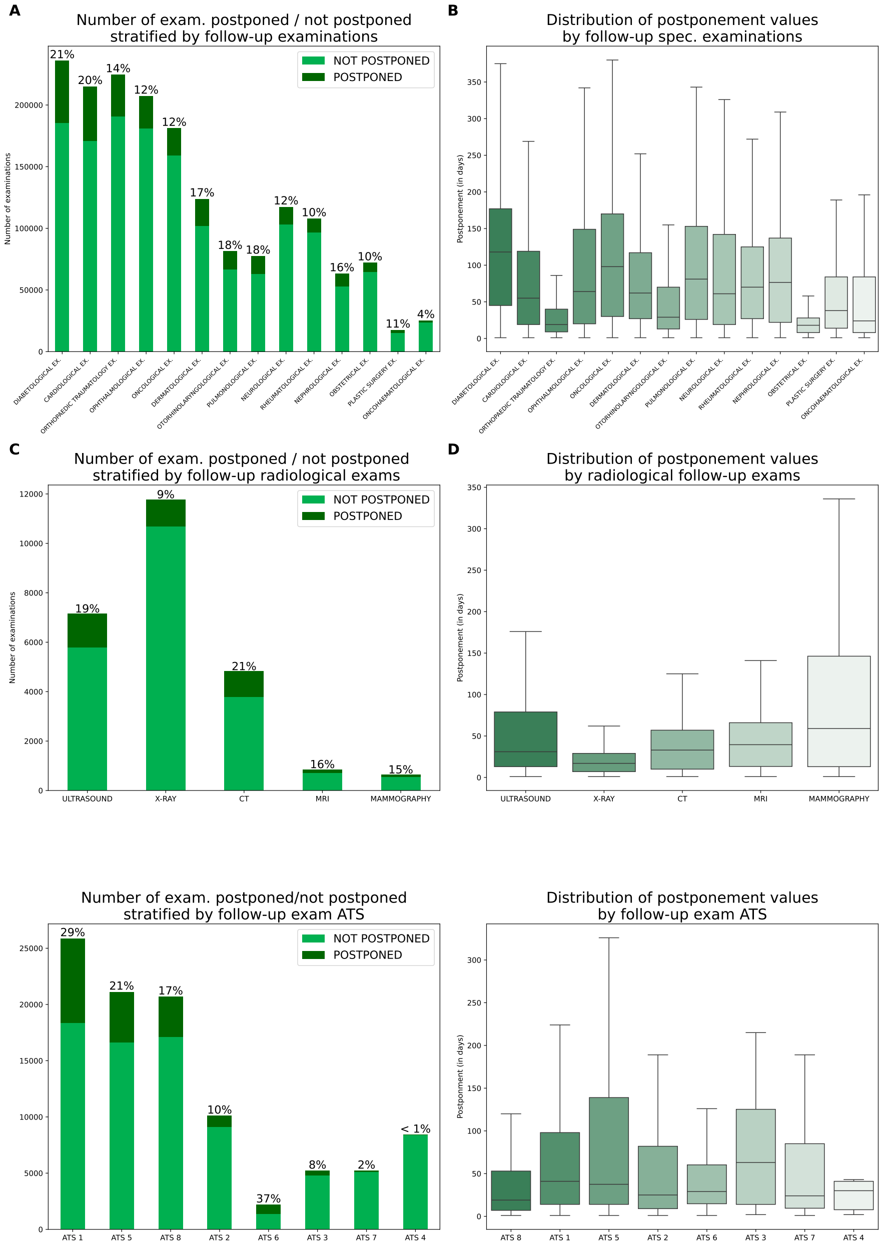

Fig. G1 Postponments of follow-up examinations by type of specialist examination, type of radiological exam and ATS

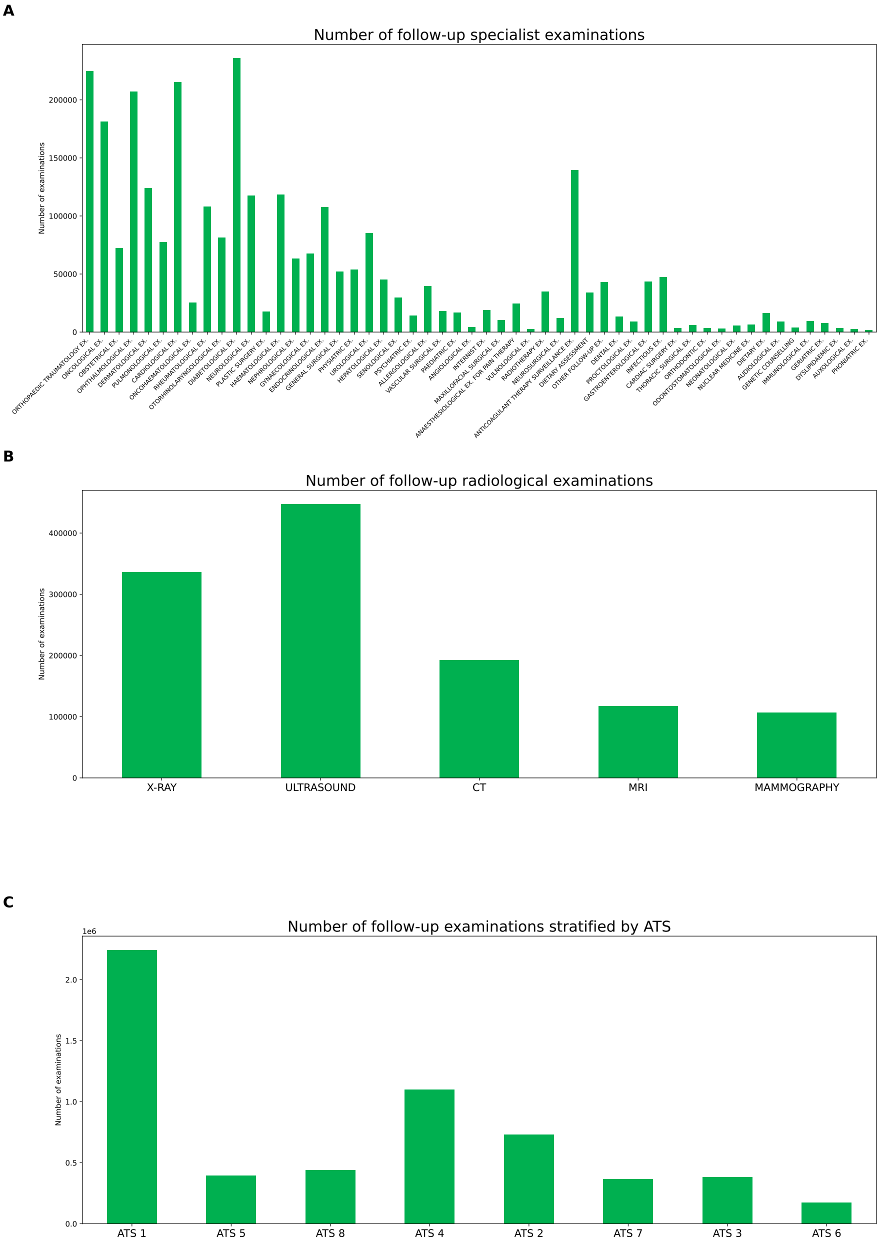

Fig. H2 Number of follow-up examinations by type of specialist examination, type of radiological exam and ATS
